# Editorialets

**Published:** 1910-01

**Authors:** 


### Editorialets.

No, Doctor; Stovaine is not a stove polish. It is *hydrochlorate of amylene.*

---

Dr. W. I. M. Smith, of Nacogdoches, lost his residence by fire December 18.

---

Dr. F. A. Maxwell, of Del Valle, was elected President of the Travis County Medical Society at its December meeting. Dr. Daniel was re-elected Delegate to State Association at Dallas in May.

---

Dr. George Dock, professor of medicine at Tulane University, New Orleans, was elected to the honorary presidency of the Section on Internal Medicine at the Sixteenth International Congress in Buda Pest.

---

Thunderstruck Indeed!—While on his travels, he was thunderstruck at receiving from his wife a telegram which ran as follows:

Twins this morning. More later.

—*December Lippincott's.*

---

The Roumanian Doctor, Jonnesco, has been demonstrating his method of using stovaine to produce spinal anesthesia at the several hospitals in New York. The *New York Medical Journal* says his results did not arouse much enthusiasm. Cocaine has been used for the purpose now about ten years; and it has not been shown that stovaine is better.

Texas Insane.—Dr. Preston, Superintendent of the Austin Insane Asylum, in his report, says: There were remaining August 31, 1908, 701 males and 636 females, total, 1337. The admissions amounted to 173 males and 155 females; total, 328. The whole number treated during the year was 1771. The number discharged was 117, and the number carried on furlough was 222, and the number of deaths was 128. The number remaining August 31, 1909, was 1391, an increase over the previous year of 54.

This is only one of the asylums. There are two others, one at Terrell and one at San Antonio, besides the epileptic colony at Abilene.

---

Pasteur Institute (Austin Insane Asylum).—This benign institution is still spreading its wings of protection over vast areas, not only of this State, but adjoining States and Territories. By referring to the report of Dr. Wilhite, physician in charge, it will be seen that 383 patients were treated during the year with one death. The comfort and satisfaction of those who seek its shrine for safety and protection is daily manifested in their bright and smiling faces as they depart from its walls with almost positive assurance of absolute freedom from the terrors of that most dread disease—hydrophobia. Had its founder, Dr. Worsham, done nothing more in a long life than establish this institution he would have been entitled to the gratitude of the people of Texas.—*Superintendent Preston's Annual Report.*

---

The Annals of Surgery Completes Its Fiftieth Volume.—The December number of the *Annals of Surgery* (Philadelphia), which completes the fiftieth volume of that journal, is worthy of more than passing notice. It is a jubilee number, and, by its size and the character of its contents, fitly marks so important an event in its history. The cosmopolitan character of the journal is seen from the list of its contributors, which comprises the leaders in surgery of England, Scotland, Denmark, France, Italy, Hawaii, Canada, and the United States.

Twenty-two articles form a number of more than four hundred pages. The illustrations, some of which are colored, are profuse, making a volume which merits the term of jubilee number. Such an event in the history of any medical journal is worthy of note.

---

Pediatrics Changes Hands.—It is with no little gratification that we learn that Dr. W. E. Fitch has purchased *Pediatrics* and will henceforth edit this well known publication. Dr. Fitch has long been connected with medical journalism as editor of *Gaillard's Southern Medicine,* and he will bring to *Pediatrics* a ripe experience both as editor and publisher. He is a graceful as well as a brilliant writer, and has contributed extensively to medical literature.

We understand that Dr. Fitch contemplates many changes in *Pediatrics,* and with a staff of collaborators which includes many of the country's foremost pediatrists, this excellent journal is certain to achieve new success in its special field. Dr. Fitch is a true Southern gentleman, and his name on the editorial page is ample assurance of the high and honorable plane on which *Pediatrics* will be conducted. If the sincere good wishes of the many friends of both *Pediatrics* and Dr. Fitch mean anything there can be no doubt of the good work that will be done in an exceedingly important branch of medicine.—*From American Medicine,* October, 1909.

